# Fractal model of anomalous diffusion

**DOI:** 10.1007/s00249-015-1054-5

**Published:** 2015-07-01

**Authors:** Lech Gmachowski

**Affiliations:** Institute of Chemistry, Warsaw University of Technology, 09-400 Plock, Poland

**Keywords:** Contracted or expanded Brownian trajectory, Supported lipid bilayer, Membrane structure, Obstacles to diffusion, Lipid molecules

## Abstract

An equation of motion is derived from fractal analysis of the Brownian particle trajectory in which the asymptotic fractal dimension of the trajectory has a required value. The formula makes it possible to calculate the time dependence of the mean square displacement for both short and long periods when the molecule diffuses anomalously. The anomalous diffusion which occurs after long periods is characterized by two variables, the transport coefficient and the anomalous diffusion exponent. An explicit formula is derived for the transport coefficient, which is related to the diffusion constant, as dependent on the Brownian step time, and the anomalous diffusion exponent. The model makes it possible to deduce anomalous diffusion properties from experimental data obtained even for short time periods and to estimate the transport coefficient in systems for which the diffusion behavior has been investigated. The results were confirmed for both sub and super-diffusion.

## Introduction

In the interior of biological cells molecules and organelles are immersed in a very crowded aqueous environment which results in specific molecular mobility. Molecule trajectories in cells are described by competing motion models which give the time dependences of the mean square of particle displacement, including free diffusion, anomalous diffusion, confined diffusion, and flow or directed motion which may result from molecular motor-driven transport. The three diffusive models can occur with directed motion, yielding more complex motion modeled by linear combinations of the dependences (Saxton and Jacobson 1997). The motion of a molecule can be classified by using a method based on Bayesian inference to calculate the a-posteriori probability of an observed trajectory on the basis of one of the models (Monnier et al. 2012). One of the mechanisms selected may be anomalous diffusion for which a single molecule trajectory, instead of being the random walk of the fractal dimension *D*_w_ = 2, is either stretched (super-diffusion, *D*_w_ < 2) or contracted (sub-diffusion, *D*_w_ > 2) (Ben-Avraham and Havlin 2000). For *D*_w_ = 1 the motion of the molecule is ballistic.

Several stochastic processes lead to anomalous diffusion; these include the continuous-time random walk, fractional Brownian motion, and Lévy flights and walks. The continuous-time random walk is a stochastic jump process in which random times occur between particle jumps with arbitrary distributions of jump lengths (Burioni et al. 2014). Fractional Brownian motion is a symmetric Gaussian process for which the second moment scales as a power of time (Jeon and Metzler 2010). Lévy flight (Viswanathan et al. 2008) is a random walk with a step-lengths probability distribution that is heavy-tailed, so the trajectory of the molecule contains occasional very long steps. In the Lévy walk the time to make a step is proportional to its length.

In cell membranes, anomalous diffusion is probably the result of both obstacles to diffusion and traps with a distribution of binding energies or escape times (Saxton and Jacobson 1997). Several detailed mechanisms were considered by Skaug et al. (2011) as the source of observed sub-diffusion: obstruction by the membrane skeleton and its bound proteins (Ritchie et al. 2003), inclusion or exclusion from lipid domains (Dietrich et al. 2002), binding to immobile traps (Saxton 2007), or a combination of these (Nicolau et al. 2007).

Sub-diffusion can be regarded as a result of coexistence of normal transport, in time periods in which a particle or molecule locally diffuses freely, and no effective transport, when the object is temporarily trapped as a result of geometrical complexity and interactions with the environment. The mean square displacement observed may, after smoothing, be described by a power-law dependence of time. This problem has been extensively studied (Burada et al. 2009; Condamin et al. 2008; Goychuk et al. 2014; Santamaria et al. 2006). Spatial restriction retards the motion of the molecule so the mean square displacement is smaller than for an unrestricted environment. The time taken to achieve a given diffusion distance is longer. Anomalous diffusion has been widely observed in the plasma membrane of biological cells, and has been used to investigate membrane organization. Sub-diffusion has been proposed as an indicator of macromolecular crowding in the cytoplasm (Weiss et al. 2004).

Super-diffusion is faster than normal diffusion. As analyzed by Stauffer et al. (2007), super-diffusion is theoretically possible in molecularly crowded environments. In biological systems, it can be the result of cellular transport processes and is observed if the diffusion is directed by a motor protein (Goychuk et al. 2014). It is believed that Lévy flights generate super-diffusion. However other mechanisms, for example fractional Brownian motion, can also lead to it (Viswanathan et al. 2008).

A moving particle or molecule follows linear segments. For a very short time the particle travels along the same segment and its movement can be regarded as ballistic (Caspi et al. 2002; Kneller 2011; Wu and Libchaber 2000), for which the fractal dimension *D*_w_ = 1. The fractal dimension then increases to achieve the asymptotic value after a very long time. Suppose that the movement can be regarded as Brownian, along a trajectory for which the fractal dimension is two. Spatial restriction in one direction, however, can retard the motion of the molecule. Wieser et al. (2007) showed equal mobility in the longitudinal and transverse directions for proteins diffusing in cellular nanotubes with saturation of the mean square displacement with time in the perpendicular direction. The measured diffusion coefficient in cellular nanotubules is lower than for unrestricted environment, and can be estimated by considering confined mobility phenomenon as early-stage Brownian motion (Gmachowski 2014).

In sub-diffusion the mechanism is different. The mean square displacement increases with time but not linearly, as observed for ordinary diffusion. This is a common property of all anomalous diffusion phenomena. The particle or molecule asymptotic trajectory is characterized by two variables, the transport coefficient *Γ* and the anomalous diffusion exponent *α*. The mean square displacement of the particle or molecule position in two dimensions, detected in experiments with long time periods, is:

1 $$ \left\langle {r^{2} } \right\rangle = 4\varGamma t^{\alpha } $$

It seems promising to describe the trajectory of a molecule by use of a modification of the scale-dependent fractal dimension method introduced by Takayasu (1982), originally for describing the transition of the trajectory fractal dimension from unity for the small scale to two for large scales. In the model proposed in this paper, the asymptotic fractal dimension of the trajectory of a molecule characterizing its long-term motion can be adjusted.

## Model

Let us analyze the fractal dimension of the random walk particle trajectory. The fractal dimension for a trajectory in fully developed Brownian motion is 2. If we consider a random walk whose mean free path is not negligible, the trajectory can be characterized by a scale-dependent fractal dimension. Observing on a scale much shorter than the mean free path, one finds the trajectory is nearly a line (Kneller 2011). Otherwise, the random walk can be reduced to the Brownian motion when analyzed on a sufficiently large scale (Bujan-Nuňez 1998; Matsuura et al. 1986; Rapaport 1984, 1985; Takayasu 1990).

The scale (*s*)-dependent fractal dimension for a random walk trajectory, given in a general form for three-dimensional space (Bujan-Nuňez 1998), is:

2 $$ D_{w} \left( s \right) = 2 - \frac{1}{1 + s/k\varLambda } $$

where *k* is a proportionality constant, being a fitting term, and *Λ* is the particle mean free path. Accordingly, *D*_w_ (*s*) varies between 1 if $s/k\varLambda \to 0$ and 2 if $s/k\varLambda \to \infty$. The larger the scale of observation, the closer is the random motion to Brownian motion.

The first term on the right side of Eq. (2) is the asymptotic fractal dimension for the Brownian trajectory. On the very small scale of observation the fractal dimension is unity, because the denominator of the second term is unity and then increases, approaching its asymptotic value when the denominator of the second term tends to infinity. The first term is thus *D*_w_, the asymptotic value of the trajectory fractal dimension, and the initial value of the fraction is *D*_w_ − 1. This imposes the form of the generalized formula.

The formula is now generalized to describe the scale-dependent fractal dimension with an adjusted asymptotic value

3 $$ D_{\text{w}} \left( s \right) = D_{\text{w}} - \frac{{D_{\text{w}} - 1}}{1 + s/k\varLambda } $$

giving the same value, 1, characteristic of ballistic motion, if $s/k\varLambda \to 0$, but a required value of *D*_w_, instead of 2, if $s/k\varLambda \to \infty$. This formula is supposed to describe the transition of the character of the particle trajectory from ballistic to that characteristic of anomalous diffusion. This approach treats the Brownian motion as a special case for which the trajectory fractal dimension tends to 2 for large scales of observation. This means that putting *D*_w_ = 2 into Eq. (3) produces Eq. (2). Putting *D*_w_ = 1 into Eq. (3) one obtains *D*_w_ (*s*) = 1, confirming the ballistic character of motion in the whole range of the observation scale.

The trajectory length depends on the scale of observation according to the fractal formula:

4 $$ \frac{{{\text{d}}\ln L\left( s \right)}}{{{\text{d}}\ln s}} = 1 - D_{\text{w}} \left( s \right) $$

Integrating with use of Eq. (3):

5 $$ \int\limits_{L\left( 0 \right)}^{L\left( r \right)} {\frac{{{\text{d}}L}}{L} = \int\limits_{0}^{r} { - \left( {D_{\text{w}} - 1} \right)\frac{{\frac{s}{k\varLambda }}}{{1 + \frac{s}{k\varLambda }}}} } \frac{{{\text{d}}s}}{s} $$

one obtains:

6 $$ \frac{r}{L\left( 0 \right)} = \frac{1}{{\left( {1 + \frac{r}{k\varLambda }} \right)^{{D_{\text{w}} - 1}} }} $$

*L*(0) is the trajectory contour length equal to the sum of the line segment lengths. The particle or molecule moves along a segment with a constant velocity *V*_0_. So the contour length can be calculated as the product of the time *t* and the mean velocity of the particle. Hence:

7 $$ \frac{1}{{\left( {1 + \frac{r}{k\varLambda }} \right)^{{D_{\text{w}} - 1}} }} = \frac{r}{{V_{0} t}} = \frac{r\tau }{\varLambda t} $$

in which the mean velocity of the particle is replaced by the mean free path of diffusing particle divided by the characteristic time, the Brownian step time:

8 $$ V_{0} = \varLambda /\tau $$

The relationship obtained is:

9 $$ \frac{r}{\varLambda }\left( {k + \frac{r}{\varLambda }} \right)^{{D_{\text{w}} - 1}} = k^{{D_{\text{w}} - 1}} \cdot \frac{t}{\tau } $$

Then, replacing *r* by $\sqrt {\left\langle {R^{2} } \right\rangle }$ one obtains the formula valid for three-dimensional space:

10 $$ \frac{{\left\langle {R^{2} } \right\rangle^{1/2} }}{\varLambda }\left( {k + \frac{{\left\langle {R^{2} } \right\rangle^{1/2} }}{\varLambda }} \right)^{{D_{\text{w}} - 1}} = k^{{D_{\text{w}} - 1}} \cdot \frac{t}{\tau } $$

Substituting in Eq. (10)

11 $$ \left\langle {R^{2} } \right\rangle = \frac{3}{2}\left\langle {r^{2} } \right\rangle $$

12 $$ \varLambda = {\sqrt {\frac{3}{2}}} \lambda $$

one obtains the formula describing the mean square displacement of the particle position in two-dimensional space 〈*r*^2^〉 as dependent on the number of steps *t*/*τ:*

13 $$ \frac{{\left\langle {r^{2} } \right\rangle^{1/2} }}{\lambda }\left( {k + \frac{{\left\langle {r^{2} } \right\rangle^{1/2} }}{\lambda }} \right)^{{D_{\text{w}} - 1}} = k^{{D_{\text{w}} - 1}} \cdot \frac{t}{\tau } $$

For short periods this formula converges to that characteristic of ballistic motion (*D*_w_ = 1):

14 $$ \left\langle {r^{2} } \right\rangle^{1/2} = \lambda \frac{t}{\tau } = v_{0} t ,$$

irrespective of the value of the fitting term *k*.

For long periods the formula obeys:

15 $$ \frac{{\left\langle {r^{2} } \right\rangle }}{{\lambda^{2} }} = k^{{\frac{{2\left( {D_{\text{w}} - 1} \right)}}{{D_{\text{w}} }}}} \cdot \left( {\frac{t}{\tau }} \right)^{{\frac{2}{{D_{\text{w}} }}}} = k^{2 - \alpha } \cdot \left( {\frac{t}{\tau }} \right)^{\alpha } $$

where *α* = 2/*D*_w_ is the anomalous diffusion exponent.

To save the universality of the formula we have to put *k* = 2. Then, for Brownian motion (*α* = 1), we obtain a known formula for the mean square displacement of the particle or molecule position in two dimensions:

16 $$ \left\langle {r^{2} } \right\rangle = 2\lambda^{2} \frac{t}{\tau } = 4Dt $$

in which the diffusion coefficient is expressed as:

17 $$ D = \frac{{\lambda^{2} }}{2\tau } $$

For long-term anomalous diffusion:

18 $$ \frac{{\left\langle {r^{2} } \right\rangle }}{{\lambda^{2} }} = 2^{2 - \alpha } \cdot \left( {\frac{t}{\tau }} \right)^{\alpha } $$

By use of Eq. (1) one can define the transport coefficient for anomalous diffusion:

19 $$ \left\langle {r^{2} } \right\rangle = 2^{2 - \alpha } \lambda^{2} \cdot \left( {\frac{t}{\tau }} \right)^{\alpha } = 4\varGamma t^{\alpha } $$

where

20 $$ \varGamma = 2^{ - \alpha } \cdot \frac{{\lambda^{2} }}{{\tau^{\alpha } }} $$

With the definition of the diffusion coefficient, expressed by Eq. (17), one obtains:

21 $$ \frac{\varGamma }{D} = \left( {2\tau } \right)^{1 - \alpha } $$

For intermediate times:

22 $$ \frac{{\left\langle {r^{2} } \right\rangle^{1/2} }}{\lambda }\left( {2 + \frac{{\left\langle {r^{2} } \right\rangle^{1/2} }}{\lambda }} \right)^{{\frac{2 - \alpha }{\alpha }}} = 2^{{\frac{2 - \alpha }{\alpha }}} \cdot \frac{t}{\tau } $$

**Fig. 1:**
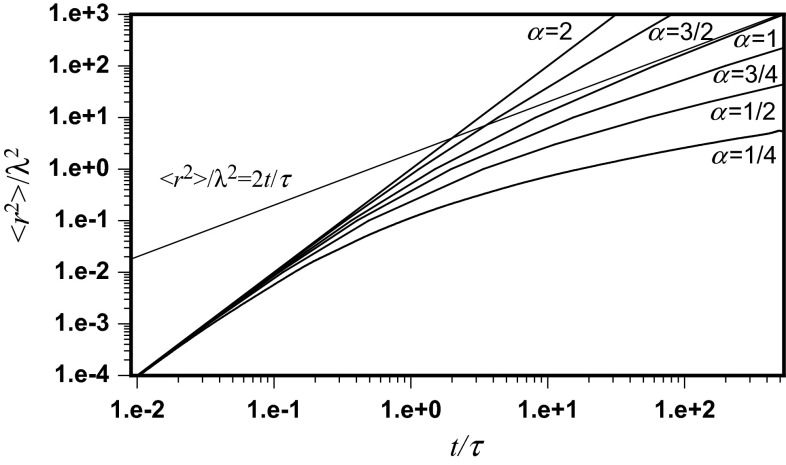
Mean square displacements of the position of the molecule, normalized by square of mean free path as dependent on normalized time, depicted for different anomalous diffusion exponents in accordance with Eq. (22). The *straight lines* correspond to the asymptotic Brownian and ballistic movements

Mean square displacements of the position of the molecule, normalized by the square of the mean free path as a function of normalized time, are depicted in Fig. 1 for different anomalous diffusion exponents. This equation, giving the interdependence of 〈*r*^2^〉/λ^2^ and *t*/*τ*, also makes it possible to draw the normalized mean square displacement of the molecule position in two dimensions 〈*r*^2^〉/4*Γt* as a function of normalized time. According to Eq. (19):

23 $$ \frac{{\left\langle {r^{2} } \right\rangle }}{{4\varGamma t^{\alpha } }} = 2^{\alpha - 2} \frac{{\left\langle {r^{2} } \right\rangle }}{{\lambda^{2} }} \cdot \left( {\frac{t}{\tau }} \right)^{ - \alpha } $$

**Fig. 2:**
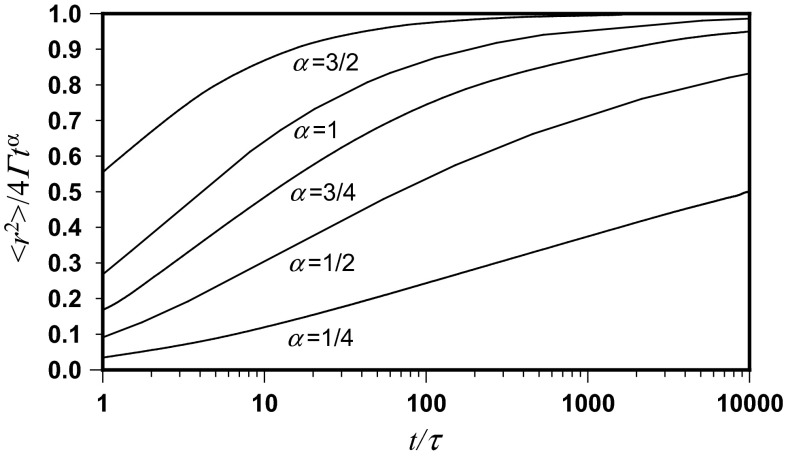
Normalized mean square displacements of the position of the molecule in two dimensions, as dependent on normalized time, depicted for different sub-diffusion exponents according to Eqs. (22) and (23)

This is done in Fig. 2 for different sub-diffusion exponents.

Equation (22) can be rearranged to:

24 $$ \left( {\frac{{\left\langle {r^{2} } \right\rangle^{1/2} }}{\lambda } \cdot \frac{\tau }{t}} \right)^{{\frac{\alpha }{2 - \alpha }}} + \left( {\frac{{\left\langle {r^{2} } \right\rangle^{1/2} }}{{2^{{\frac{2 - \alpha }{2}}} \lambda }} \cdot \left( {\frac{\tau }{t}} \right)^{{\frac{\alpha }{2}}} } \right)^{{\frac{2}{2 - \alpha }}} = 1 $$

Taking into account the definitions of the mean velocity of the particle (Eq. 14) and that of the transport coefficient (Eq. 20), one obtains:

25 $$ \left( {\frac{{\left\langle {r^{2} } \right\rangle^{1/2} }}{{v_{0} t}}} \right)^{{\frac{\alpha }{2 - \alpha }}} + \left( {\frac{{\left\langle {r^{2} } \right\rangle }}{{4\varGamma t^{\alpha } }}} \right)^{{\frac{1}{2 - \alpha }}} = 1 $$

from which the normalized mean square displacements of the molecule position can be calculated:

26 $$ \frac{{\left\langle {r^{2} } \right\rangle }}{{4\varGamma t^{\alpha } }} = \left[ {1 - \left( {\frac{{\left\langle {r^{2} } \right\rangle^{1/2} }}{{v_{0} t}}} \right)^{{\frac{\alpha }{2 - \alpha }}} } \right]^{2 - \alpha } $$

Equation (25) gives the full description of the trajectory of an anomalously diffusing molecule, irrespective of the stage of the movement. In can be used as a determinant of advancement of anomalous diffusion and may be used to determine the transport coefficient *Γ* and the anomalous diffusion exponent *α* from experimental 〈*r*^2^〉 data. This formula corresponds to the expression for ordinary diffusion (*α* = 1), previously tested for small particle movement and confined mobility in biomembranes (Gmachowski 2014). For the phenomenon in two-dimensional space it becomes:

27 $$ \frac{{\left\langle {r^{2} } \right\rangle }}{4Dt} + \frac{{\left\langle {r^{2} } \right\rangle^{1/2} }}{{v_{0} t}} = 1 $$

Equation (26) describes the time evolution of the mean square displacement of the particle or molecule position in two dimensions as dependent on the values of the transport coefficient, the anomalous diffusion exponent, and the value of the mean velocity of the particle in two dimensions. The presence of the velocity is justified by the ballistic contribution to the motion of the particle. This quantity can be determined from the diffusion coefficient by using rearranged Eq. (17):

28 $$ v_{0} = 2D/\lambda $$

Equation (22), if taken for ordinary diffusion (*α* = 1):

29 $$ \frac{{\left\langle {r^{2} } \right\rangle^{1/2} }}{\lambda }\left( {2 + \frac{{\left\langle {r^{2} } \right\rangle^{1/2} }}{\lambda }} \right) = 2 \cdot \frac{t}{\tau } $$

can serve to determine particle mean free path *λ*. Let us write this equation for two different mean square displacements of the particle position measured at two different times. The characteristic time for the two cases remains unchanged, so dividing the formulae one obtains, after rearrangement:

30 $$ \lambda = \left( {\frac{{t_{1} }}{{t_{2} }}\frac{{\left\langle {r^{2} } \right\rangle_{2} }}{{\left\langle {r^{2} } \right\rangle_{1}^{1/2} }} - \left\langle {r^{2} } \right\rangle_{1}^{1/2} } \right)/2/\left( {1 - \frac{{t_{1} }}{{t_{2} }}\frac{{\left\langle {r^{2} } \right\rangle_{2}^{1/2} }}{{\left\langle {r^{2} } \right\rangle_{1}^{1/2} }}} \right) $$

a formula serving to determine particle or molecule mean free path from data measured for ordinary diffusion.

## Comparison with experiment

Biological systems are heterogeneous. Heterogeneity is connected with non-ergodicity. Ergodic behavior is described by a stochastic process modeling anomalous diffusion under experimental investigation. So it is important to incorporate heterogeneity into modeling of biological systems (Székely and Burrage 2014). Particularly relevant for diffusion in heterogeneous media seems to be a model for heterogeneous diffusion giving an approach to non-ergodic anomalous diffusion (Cherstvy et al. 2013).

Experimental verification of ergodicity requires observation times that are sufficiently long. Skaug et al. (2011) analyzed ergodicity by comparing time-averaged and ensemble-averaged mean square displacements for anomalous diffusion measured in a lipid bilayer membrane. They showed that time-averaged mean square displacements for the longest trajectories are scattered around the ensemble averaged for the system investigated.

Skaug et al. (2011) used supported lipid bilayers to model (Chan and Boxer 2007) a real cell membrane. The researchers correlated anomalous diffusion with lipid bilayer membrane structure. The diffusion and anomalous diffusion were investigated for 1,2-dioleoyl-*sn*-glycero-3-phosphocholine (DOPC) in a supported lipid bilayer, prepared on mica, with different amounts of 1,2-distearoly-*sn*-glycero-3-phosphocholine (DSPC). Different values of the concentration of obstacles to diffusion were obtained which resulted in different values of the anomalous transport coefficient of DOPC. With no obstacles the diffusion coefficient was measured as 4.15 μm^2^/s. Increasing of the area fraction of the obstacles reduced both the transport coefficient and the anomalous diffusion exponent. The authors presented experimental data in the form of time dependences of the mean square displacements measured for several different area fractions of obstacles.

By using values of the mean square displacements measured for ordinary diffusion at limiting experimental times of 0.035 and 0.14 s, the value of the mean free path *λ* = 0.0559 μm was calculated by use of Eq. (30). Then one Brownian step time τ = 3.77 × 10^−4^ s was computed by use of Eq. (17).

The mean velocity of the molecule in two dimensions, appearing in Eq. (26), is expressed by *D*, Γ and *α*. To achieve this we combine Eqs. (17, 21, 28) to obtain:

31 $$ v_{0} = 2\sqrt D \left( {\frac{\varGamma }{D}} \right)^{{\frac{1}{{2\left( {\alpha - 1} \right)}}}} $$

**Fig. 3:**
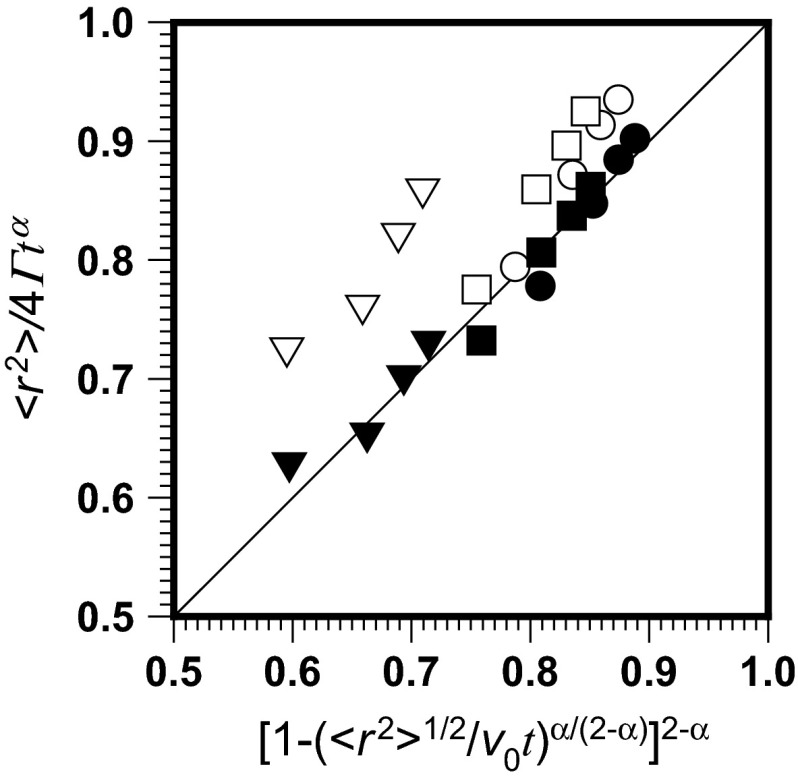
Graphical representation of Eq. (26) with *v*_0_ given by Eq. (31), fitted by experimental results of mean square displacement normalized by use of the originally calculated data for DOPC transport in supported lipid bilayers (Skaug et al. 2011): *open circles*, *α* = 0.86, *Γ* = 1.55; *open squares*, *α* = 0.77, *Γ* = 0.78; *open inverse triangles*, *α* = 0.56, *Γ* = 0.16. The corresponding *filled symbols* are drawn for best fit data obtained by use of Eq. (21): *filled circles*, *α* = 0.871, *Γ* = 1.64; *filled squares*, *α* = 0.780, *Γ* = 0.854; *filled inverse triangles*, *α* = 0.573, *Γ* = 0.193

Equation (26) with *v*_0_ given by Eq. (31), is fitted by experimental results for mean square displacement for times 0.035, 0.07, 0.105, and 0.14 s, normalized by $4\varGamma t^{\alpha }$ using originally calculated (Skaug et al. 2011) data of *α* = 0.86, *Γ* = 1.55; *α* = 0.77, *Γ* = 0.78; *α* = 0.56, *Γ* = 0.16. This is represented by open symbols in Fig. 3. The corresponding filled symbols are drawn for best fit data obtained by use of Eq. (21) with the determined value of $\tau = 3.77 \times 10^{ - 4}$ s. They are *α* = 0.871, *Γ* = 1.64; *α* = 0.780, *Γ* = 0.854; *α* = 0.573, and *Γ* = 0.193.

Although the original values are only slightly lower than those calculated by use of the proposed method, quite different location of points represented by open symbols and filled symbols may be observed in Fig. 3. The filled symbols are much closer to the model line, this is a result of a more precise determination of transport variables by use of proposed method. Very similar results, *α* = 0.862, *Γ* = 1.54; *α* = 0.773, *Γ* = 0.812; *α* = 0.582, and *Γ* = 0.206, can be obtained by using only the mean square displacement for the shortest time, 0.035 s. The corresponding values of the normalized mean square displacement 〈*r*^2^〉/4*Γt*^*α*^ are 0.803, 0.752, and 0.608, which means that the proposed method enables effective analysis of short-term experimental data.

The calculated values of the mean velocity of the molecule in two dimensions are all 148 μm/s, which corresponds to *λ* = 0.0559 μm and one Brownian step time *τ* = 3.77 × 10^−4^ s, both computed previously from data measured for ordinary diffusion. The values of the mean velocity computed by use of Eq. (31) using originally calculated (Skaug et al. 2011) data of *α* = 0.86, *Γ* = 1.55; *α* = 0.77, *Γ* = 0.78; *α* = 0.56, *Γ* = 0.16 are 137, 154, and 165 μm/s, i.e. almost the same.

**Fig. 4:**
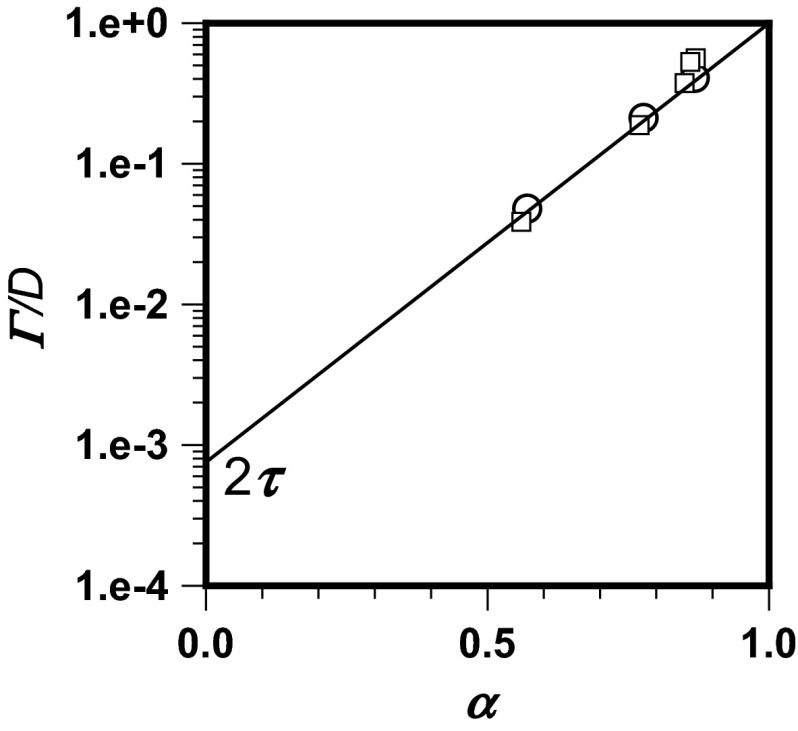
Normalized transport coefficient of DOPC in supported lipid bilayers (Skaug et al. 2011) as a function of the anomalous diffusion exponent calculated by use of Eq. (21) and using Eqs. (28) and (30) to determine the one step time *τ* (*solid line*), fitted with originally calculated values of *α* and *Γ* (*open squares*) and those chosen to fit Eq. (26) (*open circles*)

The values of transport variables determined by use of the proposed method are obtained by use of Eq. (21). The results are depicted in Fig. 4, which shows all pairs of transport variables *α* and *Γ* reported by Skaug et al. (2011). Agreement of reported results with the model line is good.

Complete results for super-diffusion in biological systems are not available in the literature. To demonstrate the reliability and potential usefulness of the proposed model for a wider range of anomalous diffusion exponents, results obtained by Li et al. (2006) for one-dimensional sub and super-diffusive molecular displacements in disordered porous media were used. Hydrodynamic dispersion of water flowing through porous glass with nominal pore sizes in the range 100–160 μm was analyzed. The character of the anomalous diffusion behavior depended on the flow velocity through the porous medium. Crossover was observed from sub-diffusive mean square displacement, *α* = 0.84, in the absence of hydrodynamic flow, to a super-diffusive, almost ballistic power law, *α* = 1.95, at the highest flow rates.

Let us write Eq. (18) for two different mean square displacements of the molecule position measured at the same time for anomalous and normal diffusion. The molecule mean free path for the two cases remains unchanged, so dividing the formulae one obtains, after rearrangement:

32 $$ \tau = \left( {\left\langle {x^{2} } \right\rangle /\left\langle {x^{2} } \right\rangle_{Br} } \right)^{{\frac{1}{1 - \alpha }}} \cdot \frac{t}{2} $$

where the displacements in two dimensions are replaced by that in one dimension. From the experimental plot reported by Li et al. (2006) giving the time dependence of the mean square displacement, it is possible to show that the mean square displacement ratio is 8.5 for *α* = 1.95 and *α* = 1 and time equal to 0.3 s. The calculated Brownian step time is *τ* = 1.58 × 10^−2^ s.

The values of the transport coefficient normalized by the diffusion coefficient were then calculated from the plot for the same time and Eq. (19) rearranged for one-dimensional displacement to give:

33 $$ \left\langle {x^{2} } \right\rangle = 2\varGamma t^{\alpha } $$

and

34 $$ \left\langle {x^{2} } \right\rangle_{\text{Br}} = 2Dt $$

from which:

35 $$ \frac{\varGamma }{D} = \left\langle {x^{2} } \right\rangle /\left\langle {x^{2} } \right\rangle_{\text{Br}} t^{1 - \alpha } $$

The values determined are 0.58 s^0.16^ for *α* = 0.84 and 27 s^−0.95^ for *α* = 1.95, whereas the diffusion coefficient calculated from Eq. (34), *D* = 2.0 × 10^−9^ m^2^/s, is only slightly lower than the molecular diffusivity of bulk water (2.3 × 10^−9^ m^2^/s).

To obtain a universal coordinate system for anomalous diffusion variables, in which the variables could be compared for different characteristic times, Eq. (21) is rearranged to give:

36 $$ \frac{\varGamma }{D} \cdot \tau^{\alpha - 1} = 2^{1 - \alpha } $$

**Fig. 5:**
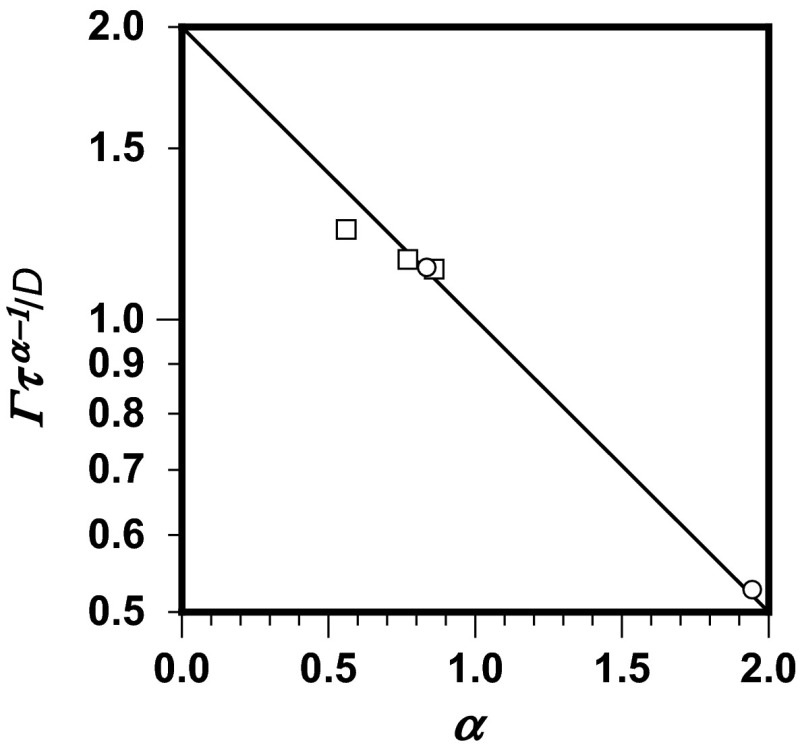
Graphical representation of Eq. (36) (*solid line*) fitted with the originally calculated values of *α* and *Γ* for DOPC transport in supported lipid bilayers (Skaug et al. 2011; *τ* = 3.77 × 10^−4^ s; *open squares*) and the values deduced from reported data (Li et al. 2006) for displacement of water molecules in disordered porous glass (*τ* = 1.58 × 10^−2^ s; *open circles*)

The resulting Fig. 5 presents all the experimental values originally calculated (Skaug et al. 2011) and deduced from reported data (Li et al. 2006), covering both the sub-diffusion and super-diffusion ranges.

All the calculations discussed in this paper confirm the accuracy of original estimate and provide an effective fractal model for the trajectory of particles or molecules diffusing anomalously. The model takes into account ballistic motion, which is essential at the very beginning of the motion, and the time evolution of the trajectory character. The model increases the precision of the transport variables obtained for anomalous diffusion.

## Discussion and conclusions

This fractal model of Brownian particle motion makes it possible to describe the trajectory of a molecule diffusing anomalously if the asymptotic fractal dimension of the trajectory is regarded as an adjustable variable. Except for the power dependence valid for long-term diffusion, the model describes the early stage of the transition from ballistic to sub or super-diffusive motion. The formula obtained (Eq. 26) makes it possible to derive the values of the transport coefficient and the anomalous diffusion exponent from experimental data even if the data are measured in the short term, when the power dependence (Eq. 1) is still not fully applicable.

The data describing anomalous diffusion are connected with those describing normal diffusion. The transport coefficient normalized by the diffusion constant is the double Brownian step time to the power of one minus the anomalous diffusion exponent (Eq. 21). This is a functional dependence enabling more precise derivation of the anomalous diffusion variables from experimental data.

The originally obtained anomalous diffusion variables, both the transport coefficient and the anomalous diffusion exponent, are slightly lower than those calculated by use of the proposed method. This is because of the time period over which the experimental data were measured, too short to safely use the power dependence (Eq. 1). Small differences in the anomalous diffusion variables result, however, in quite different location of points in Fig. 3. More precise determination of the transport variables leads to reduction of the mutual distance. The points calculated by use of Eq. (21) are much closer to the model line.

The proposed method is applicable to data obtained in short time periods. Transport variables calculated solely by use of experimental data for mean square displacement in the shortest times are in good agreement with those determined for longer time periods.

As already mentioned, and confirmed experimentally, the model presented in this paper is not restricted to sub-diffusion. It describes super-diffusive trajectories up to ballistic trajectories for which *α* = 2. The corresponding form of Eq. (20) is:

$$ \varGamma = \frac{{\lambda^{2} }}{{4\tau^{2} }} = \frac{{v_{0}^{2} }}{4} $$

Hence Eq. (19) becomes:

$$ \left\langle {r^{2} } \right\rangle^{1/2} = v_{0} t = \lambda \cdot t/\tau $$

which one would expect for ballistic motion. The root mean square displacement is the mean free path multiplied by the number of steps.

Statistical models and methods lead to similar power-law characteristics for anomalous diffusion, for example continuous time random walks (Tejedor and Metzler 2010; Neusius et al. 2009), fractional Langevin Brownian motion (Jeon and Metzler 2010), and mixed models with different trapping (Miyaguchi and Akimoto 2015). Recent models combined Bayesian inference (Monnier et al. 2012) with the over-damped Langevin equation in which spatially varying friction is used, reflecting the heterogeneity of the plasma membrane on the full cell scale. In that way variables for different scales (Masson et al. 2014) were obtained.

Models describing the ballistic-sub-diffusive transition are of special interest for experimenters interested in single-particle-tracking. Jeon and Metzler (2010) presented an exact solution to the fractional Langevin equation in the form of the time dependence of mean-squared displacement, and showed a transition from short-term ballistic motion to long-term anomalous diffusion. In this solution the generalized Mittag–Leffler function is used. The function obtained is depicted in a plot. The observed transition from the ballistic to sub-diffusion region is rather narrow and spans no more than one order of magnitude of time. The time average mean-square displacement for fractional Brownian–Langevin motion presented by Deng and Barkai (2009) has a similar property.

The equation proposed in this paper, and the form which is simpler to use, has a wider transition which spans approximately three orders of magnitude of time for ordinary diffusion of Brownian particles (Gmachowski 2013, 2014; Pusey 2011). This wide transition has been confirmed by use of the experimental data analyzed in this work. The wideness of the transition and, hence, the shape of the time dependence of the mean square displacement of the molecule, is of fundamental importance, because the experimental trajectories are in the range of the transition.

Analysis of experimental data shows that the fractal model of molecule trajectories is sufficient to describe anomalous diffusion phenomena. The model trajectory consists of equal segments which can be either contracted or stretched, modeling sub-diffusive or super-diffusive phenomena. Therefore, it can be regarded as a new approach to anomalous diffusion which is a simple alternative to current models with distributions of step lengths. The transition of the character of the motion from ballistic to anomalous diffusion is described by the time evolution of the trajectory fractal dimension. This can be regarded as a new aspect of the modeling of anomalous diffusion which results in the wider transition observed experimentally.

A universal coordinate system has been derived for anomalous diffusion in which the anomalous diffusion variables can be compared for different characteristic times in the full range of the anomalous diffusion exponent. This can be helpful for interpretation of experimental data, especially those obtained for short periods of time. This also enables estimation of the transport coefficient for systems for which the diffusion behavior has been investigated.

## List of symbols

*D*: Diffusion coefficient (μm^2^/s)
*D*_w_: Differential fractal dimension (–)
*L*: Trajectory length (μm)
*s*: Scale of observation (μm)
*t*: Time (s)
*V*_0_: Mean velocity of the particle or molecule (μm/s)
*v*_0_: Mean velocity of the particle or molecule in two dimensions (μm/s)
〈*r*^2^〉: Mean square displacement of the particle or molecule position in two dimensions (μm^2^)
〈*R*^2^〉: Mean square displacement of the particle or molecule position in three dimensions (μm^2^)
〈*x*^2^〉: Mean square displacement of the particle or molecule position in one dimension (μm^2^)
*α*: Anomalous diffusion exponent (–)
*Γ*: Transport coefficient (μm^2^/s^α^)
*λ*: Particle or molecule mean free path in two dimensions (μm)
*Λ*: Particle or molecule mean free path in three dimensions (μm)
*τ*: Brownian step time (s)
